# Digital phenotyping of stress responsive suicidal ideation in healthy individuals: an opportunity for prevention

**DOI:** 10.1192/j.eurpsy.2025.113

**Published:** 2025-08-26

**Authors:** M. A. Oquendo

**Affiliations:** Psychiatry, University of Pennsylvania, Philadelphia, United States

## Abstract

**Background:**

Decedents without known mental disorders constitute 5%-40% of suicides and epidemiologic studies show that 20% of those with a history of suicidal behavior (SB) may be free of psychiatric disorders. This implies that suicide ideation (SI) may occur in the psychiatrically healthy volunteers (HVs), providing an opportunity for preventing suicide and SB. Yet, studies of HVs have not focused on the occurrence of SI, nor has SI in HVs been characterized, especially in relation to the occurrence of stress. If SI is not rare in HVs, current approaches to managing suicide risk in medical settings may be too limited to identify psychiatrically healthy individuals at risk for suicide.

**Aims & Objectives:**

We recruited HVs and major depressive disorder (MDD) patients to test two hypotheses: (1) some HVs, screened to exclude those meeting lifetime criteria for Axis I or II disorders and with no personal or family history of SB, would endorse SI on Ecological Momentary Assessment (EMA); and (2) HVs’ SI scores would be lower than those of MDD patients. In addition, we investigated whether HV and MDD groups had different responses to stressors in terms of SI.

**Method:**

HVs and MDD patients were recruited through advertisements: some patients were recruited in the emergency department. Participants provided 7 days of ecological momentary assessment (EMA) data about SI and stressors. EMA data was collected in six 2-hour epochs during a 12-hour period selected to match participant wake hours. Longitudinal mixed effects logistic regression models compared HV and patient SI scores and frequency of stressors. Mixed effects linear regression models compared HVs’ and patients’ SI score change from the previous epoch’s SI score when each stressor occurred.

**Results:**

HVs (n=42) reported less frequent (p<0.001) and less intense SI (p<0.003) than patients (n=80). HVs did endorse SI and/or SI-related items in 44% of EMA epochs, endorsing actual SI items, as opposed to SI related items (lack of wish to live) in 25% of epochs with non-zero SI scores. For 7 of 8 stressors, patients reported stressors more often than HVs (all p<0.001) and responded to them with greater SI (0.0001<p<0.0472). HVs reported statistically significant SI increases only in response to neglect (p<0.0147), indicating HVs were relatively resilient to other stressors.

**Discussion & Conclusion:**

The occurrence of SI and SB among psychiatrically healthy individuals has been relatively neglected. However, SI does occur in this population. Although HVs demonstrated more modest SI increases than patients in response to stressors, the reports of SI in 44% of epochs by HVs suggest that focusing solely on individuals with mental disorders when screening for suicide risk may be too narrow an approach.

**Disclosure of Interest:**

M. Oquendo, MD, PhD earned royalties from: Research Foundation for Mental Hygiene, Inc. - Columbia Suicide Scale, Conflict with: Honoraria - Alkermes - Grant Reviews, Honorarium.

